# Supplementary material for: Quantitative glycoproteomics of human milk and association with atopic disease
Source: PLoS One. 2022 May 13;17(5):e0267967. doi: 10.1371/journal.pone.0267967 (PMC9106177; doi:10.1371/journal.pone.0267967)
Supplement: Attachment — Submitted filename: Response to reviewers.docx [file pone.0267967.s007.docx]

**Response to reviewers**

**Journal Requirements:**

**When submitting your revision, we need you to address these additional requirements.**

**1. Please ensure that your manuscript meets PLOS ONE's style requirements, including those for file naming. The PLOS ONE style templates can be found at**

[**https://journals.plos.org/plosone/s/file?id=wjVg/PLOSOne_formatting_sample_main_body.pdf**](https://urldefense.proofpoint.com/v2/url?u=https-3A__journals.plos.org_plosone_s_file-3Fid-3DwjVg_PLOSOne-5Fformatting-5Fsample-5Fmain-5Fbody.pdf&d=DwQGaQ&c=4sF48jRmVAe_CH-k9mXYXEGfSnM3bY53YSKuLUQRxhA&r=R44Ma_DTBsJg20mFPbixYkhh4janOIAtHSN7N0klXKf1pW1dSPrz7PKi6ZbsUR6H&m=NOL768a6tHHAWAxiW1CsSbVMJWDBNy43q4xHvV3AVq0&s=_cWvViyZeB04qcQW86-NeGPMzvehwvM0_muKt0OIXyg&e=) **and**

[**https://journals.plos.org/plosone/s/file?id=ba62/PLOSOne_formatting_sample_title_authors_affiliations.pdf**](https://urldefense.proofpoint.com/v2/url?u=https-3A__journals.plos.org_plosone_s_file-3Fid-3Dba62_PLOSOne-5Fformatting-5Fsample-5Ftitle-5Fauthors-5Faffiliations.pdf&d=DwMGaQ&c=4sF48jRmVAe_CH-k9mXYXEGfSnM3bY53YSKuLUQRxhA&r=R44Ma_DTBsJg20mFPbixYkhh4janOIAtHSN7N0klXKf1pW1dSPrz7PKi6ZbsUR6H&m=NOL768a6tHHAWAxiW1CsSbVMJWDBNy43q4xHvV3AVq0&s=0SkKNG8e7PGKJNjdAxTKtapam-vuPFaWUpbSAb6GBeE&e=)

The appropriate changes have now been made.

**2. In your Data Availability statement, you have not specified where the minimal data set underlying the results described in your manuscript can be found. PLOS defines a study's minimal data set as the underlying data used to reach the conclusions drawn in the manuscript and any additional data required to replicate the reported study findings in their entirety. All PLOS journals require that the minimal data set be made fully available. For more information about our data policy, please see** [**http://journals.plos.org/plosone/s/data-availability**](https://urldefense.proofpoint.com/v2/url?u=http-3A__journals.plos.org_plosone_s_data-2Davailability&d=DwMGaQ&c=4sF48jRmVAe_CH-k9mXYXEGfSnM3bY53YSKuLUQRxhA&r=R44Ma_DTBsJg20mFPbixYkhh4janOIAtHSN7N0klXKf1pW1dSPrz7PKi6ZbsUR6H&m=NOL768a6tHHAWAxiW1CsSbVMJWDBNy43q4xHvV3AVq0&s=5vGi27JbTP2pdmzGNTo-ZCg46zNzkT5ny7R3B8ykDS8&e=)**.**

**Upon re-submitting your revised manuscript, please upload your study’s minimal underlying data set as either Supporting Information files or to a stable, public repository and include the relevant URLs, DOIs, or accession numbers within your revised cover letter. For a list of acceptable repositories, please see** [**http://journals.plos.org/plosone/s/data-availability#loc-recommended-repositories**](https://urldefense.proofpoint.com/v2/url?u=http-3A__journals.plos.org_plosone_s_data-2Davailability-23loc-2Drecommended-2Drepositories&d=DwQGaQ&c=4sF48jRmVAe_CH-k9mXYXEGfSnM3bY53YSKuLUQRxhA&r=R44Ma_DTBsJg20mFPbixYkhh4janOIAtHSN7N0klXKf1pW1dSPrz7PKi6ZbsUR6H&m=NOL768a6tHHAWAxiW1CsSbVMJWDBNy43q4xHvV3AVq0&s=msk2uJOgMzy8NBDnbf7N9NgBMxGKuKS6JitF617ANj0&e=)**. Any potentially identifying patient information must be fully anonymized.**

**Important: If there are ethical or legal restrictions to sharing your data publicly, please explain these restrictions in detail. Please see our guidelines for more information on what we consider unacceptable restrictions to publicly sharing data:** [**http://journals.plos.org/plosone/s/data-availability#loc-unacceptable-data-access-restrictions**](https://urldefense.proofpoint.com/v2/url?u=http-3A__journals.plos.org_plosone_s_data-2Davailability-23loc-2Dunacceptable-2Ddata-2Daccess-2Drestrictions&d=DwQGaQ&c=4sF48jRmVAe_CH-k9mXYXEGfSnM3bY53YSKuLUQRxhA&r=R44Ma_DTBsJg20mFPbixYkhh4janOIAtHSN7N0klXKf1pW1dSPrz7PKi6ZbsUR6H&m=NOL768a6tHHAWAxiW1CsSbVMJWDBNy43q4xHvV3AVq0&s=jNYka20CNZxJdcqX8WSS7sGHnzuTjwzZFLPYRrIE7TA&e=)**. Note that it is not acceptable for the authors to be the sole named individuals responsible for ensuring data access.**

**We will update your Data Availability statement to reflect the information you provide in your cover letter.**

In our Data Availability statement we have mentioned that “The data underlying this article are available at the ProteomeXchange Consortium (<http://proteomecentral.proteomexchange.org>) via the PRIDE partner repository [24] and can be accessed with the dataset identifier PXD026644.”.

This data can currently be accessed with a reviewer account (username: [reviewer_pxd026644@ebi.ac.uk](mailto:reviewer_pxd026644@ebi.ac.uk), password: O7txVqOc) but will be made public if our manuscript is accepted for publication.

**3. We note that you have stated that you will provide repository information for your data at acceptance. Should your manuscript be accepted for publication, we will hold it until you provide the relevant accession numbers or DOIs necessary to access your data. If you wish to make changes to your Data Availability statement, please describe these changes in your cover letter and we will update your Data Availability statement to reflect the information you provide.**

We have previously provided the relevant accession number/dataset identifier in our Data Availability statement.

**5. Review Comments to the Author

Please use the space provided to explain your answers to the questions above. You may also include additional comments for the author, including concerns about dual publication, research ethics, or publication ethics. (Please upload your review as an attachment if it exceeds 20,000 characters)**

**Reviewer #1: Overall, this is a well performed study and is well described. I think that it provides a nice set of information for the field which may lead to future research examining the potential impact of the associations between site-specific glycosylation of milk proteins and allergy.

Abstract
32: N-glycopeptide should be N-glycoprotein—as you are using tryptic peptides to identify the glycoproteins.**

This has been amended.

**34: same comment. Should be glycoprotein, not glycopeptide profiles. Saying glycopeptide implies that you studied the naturally occurring glycopeptides rather than the trypsin-produced peptides.**

This has been amended. We thank the reviewer for this observation.

**Intro:
93: you are actually comparing N-glycoprotein profiles (via tryptic N-glycopeptides), not N-glycopeptide profiles—the N-glycopeptides here are created in your study, not a natural phenomenon.**

This has also been amended.

**Results
Table 2: max fold change and P-value numbers should have decimal points as periods, not commas. Same for Table 3.**

This has now been fixed.

**Method
424: Should give time and temperatures for centrifugation steps.**

These have been added.

**433: iodoacetamide should not be capitalized.**

This has been fixed.

**Statistics: It seems that a correction for multiple testing should be applied (each test increases the likelihood of a false positive). I do not see one listed.**

This has now been provided in Supplementary Tables 2, 4, and 5.

**Reviewer #2: Data interpretation: The abstract is overstretched. “These four glycopeptides may have a protective effect against the development of atopic disease. Our findings indicate that the differential glycosylation of milk proteins may affect the development of atopic disease, something previously uninvestigated.” As the authors do present a mere association, yet no mechanistic hypothesis, and also no experimental support for a causal relationship whatsoever, these final sentences of the abstract appear poorly supported. Please adjust.**

We have now stated that these four glycopeptides may be associated with the development of atopic disease and that the findings of our study suggest that the differential glycosylation of milk proteins could be linked to atopic disease.

**Data interpretation: what are the differences between the milk samples? Please try and distill from the data some major changes: how is site-specific glycosylation different, is e.g. sialylation up or down, are there differences in fucosylation?**

We understand what the reviewer is asking, but due to the nature of this study, these kinds of conclusions are unable to be drawn from the data. In order to answer questions regarding for example the major changes in fucosylation or sialylation, it would have been better to release the sugars and analyze them to give an idea of the major global changes in glycosylation. When we talk about site-specific changes as they are presented in the tables, they are site- and protein-dependent, and it is therefore hard to derive any general conclusions regarding overall changes between the groups.

**To which extent are the differences in glycopeptide signals caused by differences in the expression of proteins, versus differences in site-specific glycosylation? An integrated proteomic and glycoproteomic analysis of the data would be necessary.**

This is a very good question. Nowadays, the current workflows available enable integrated analyses, but as these samples were collected and analyzed at a time when this option was unavailable, we are unfortunately unable to provide it for this study. However, an integrated proteomic and glycoproteomic analysis is something that we definitely plan on including in a follow-up study.

**Glycopeptide identification: The authors mention that MS/MS assignments were manually checked, but they do not display any of the MS/MS spectra. Please provide assigned MS/MS spectra as supplementary data for all de novo assignments and for all more exotic compositions and proposed structures.**

We have provided these in the PRIDE entry, which can currently be accessed with reviewer details and will be made public if this manuscript is accepted for publication. However, as mentioned in the manuscript, the proposed structures are those that are the best matches when compared to the database. The composition of the glycan is calculated as the one that best matches the mass that is left after the mass of the peptide is deducted from the total glycopeptide mass. The proposed structures should therefore be considered as potential structures in case anyone would be interested in verifying their structures by other means (such as enzymatically). In this field, it has been a long-standing tradition to rely on the total mass and present glycan structures and peptide sequences that will fit the total mass instead of validating the compositions by fragmentation. However, despite not verifying the proposed structures with a method like this, our study includes a further step in the analysis, as we also fragment the glycopeptides and fit the peptides and glycans on these fragments instead of relying on total mass alone.

**Figure 1: the H3N3S1 glycan structure is not credible, with the sialic acid linked to GlcNAc; please adjust**

The reviewer is correct, although this structure is possible as a monoantennary structure. This structure has been removed from Figure 1. We thank the reviewer for noticing this mistake.

**Figure 2: what is the structural basis for assigning fucoses to the core, to the antennary GlcNAc, or antennary galactose; please provide support for these assignments, same for figure 3.**

Core fucosylation takes place in the most innermost GlcNAc residue of an N-linked glycan. When a glycopeptide is fragmented and core fucosylated, we can often see fragments when the peptide is not fragmented but various monosaccharides are lost from the antennae. This most likely happens when the collision energy is ramped, as was done in this study, and different amounts of monosaccharides are lost from the parent ion. When it happens that there is only an intact peptide and a fucosylated N-core, or one or more monosaccharides have been fragmented from the N-core, it means that the only place the fucose residue can be located is the innermost GlcNAc (=core fucosylation). Sometimes only one fragment is found, and sometimes plenty of fragments. We have manually curated the spectra and looked for these fragments. This works only as a positive identification, and if no such fragments are found, we cannot be absolutely certain if it is a neutral loss of a core fucosylation or a branch fucosylation. In the case of two fucose residues in the composition, the other must be in the branch since there can be only one fucose residue in the N-core. The fragments are annotated in the PRIDE entry “Summary_MS2_Fragments” table.

**Quantification: quantifying each charge state separately does not make sense, as the aim is to quantify glycopeptides, not MS signals. Hence, please sum up the different charge states before quantification, or rely on the most intense one.**

We have preferred to leave them as ions as they are fragmented separately—they come out at the same time from the reverse phase column but are analyzed independently of each other. In our opinion, they serve as positive controls if the results are similar enough. As seen in the examples provided below, the fold changes for the identical compositions are very similar, as is their significance as analyzed by the Benjamini-Hochberg method. This is why we prefer to leave them in this format.

| 1116,2 | 4 | PIGR_HUMAN | 421 | LSLLEEPGNGTFTVILNQLTSR | S1H5N4F1 | SHNH(F(H)NH)HNN | 42,1 | 1,5 | A | B | 6,51E-02 | not significant |
| --- | --- | --- | --- | --- | --- | --- | --- | --- | --- | --- | --- | --- |
| 1488,0 | 3 | PIGR_HUMAN | 421 | LSLLEEPGNGTFTVILNQLTSR | S1H5N4F1 | SHNH(F(H)NH)HNN | 65,3 | 1,9 | A | B | 2,94E-02 | not significant |

| 1058,9 | 5 | TRFL_HUMAN | 156 | TAGWNVPIGTLRPFLNWTGPPEPIEAAVAR | S1H5N4F1 | SHNH(HNH)HN(F)N | 54,2 | 1,5 | A | B | 2,74E-01 | not significant |
| --- | --- | --- | --- | --- | --- | --- | --- | --- | --- | --- | --- | --- |
| 1323,4 | 4 | TRFL_HUMAN | 156 | TAGWNVPIGTLRPFLNWTGPPEPIEAAVAR | S1H5N4F1 | SHNH(HNH)HN(F)N | 69,2 | 1,8 | A | B | 2,02E-01 | not significant |

| 1152,8 | 4 | PIGR_HUMAN | 421 | LSLLEEPGNGTFTVILNQLTSR | S1H5N4F2 | SHNH(FHNH)HN(F)N | 85,6 | 2,5 | A | B | 1,16E-01 | not significant |
| --- | --- | --- | --- | --- | --- | --- | --- | --- | --- | --- | --- | --- |
| 1536,7 | 3 | PIGR_HUMAN | 421 | LSLLEEPGNGTFTVILNQLTSR | S1H5N4F2 | SHNH(FHNH)HN(F)N | 97,3 | 1,4 | A | B | 2,49E-02 | not significant |

| 953,4 | 4 | CASA1_HUMAN | 69 | NESTQNCVVAEPEK | S1H5N4F2 | SHNH(FHNH)HN(F)N | 51,7 | 2,3 | A | B | 1,54E-02 | significant |
| --- | --- | --- | --- | --- | --- | --- | --- | --- | --- | --- | --- | --- |
| 1270,8 | 3 | CASA1_HUMAN | 69 | NESTQNCVVAEPEK | S1H5N4F2 | SHNH(FHNH)HN(F)N | 57,0 | 2,8 | A | B | 7,99E-03 | significant |

**The quantification needs some clarification: For example, it is unclear how the fold change was calculated, as the tables have a column headed Max fold change. What is a Max fold change? I searched the manuscript for explanation, but could not find it.**

The max fold change comes from the Progenesis software where the data was quantified and is connected to the highest and lowest mean conditions. The fold change here is always positive and the highest or lowest mean indicates in which group the levels of a glycopeptide were lower or higher. It is a useful way to tell where the largest change in abundance between the groups is found. An explanation has been added to the legend for Table 2.

**In line with this comment, please provide comprehensive legends for all suppl. Tables to guide the reader in assessing the data.**

Legends for all supplementary tables are provided in the manuscript after the references.
