# Supplementary material for: Quantitative glycoproteomics of human milk and association with atopic disease
Source: PLoS One. 2022 May 13;17(5):e0267967. doi: 10.1371/journal.pone.0267967 (PMC9106177; doi:10.1371/journal.pone.0267967)
Supplement: Attachment — Submitted filename: Response to reviewers.docx [file pone.0267967.s008.docx]

**Replies to reviewer comments**

**We thank the reviewers for their suggestions. We have addressed all the comments as followes.**

**Reviewer #1: All changes requested have been made accurately. The manuscript is now ready for publication in the journal.**

**Reviewer #2: The authors have considerably improved the manuscript by toning down on some statements, and including more information in e.g figure and table legends. A lot of the key data of the paper are, however, not sufficiently accessible, and I therefore propose a major revision of the manuscript, to make some of the conclusions more substantiated, make the paper more comprehensible, and ultimately increase its impact.**

**1. Display of MS/MS data. The authors indicate that MS/MS data are made online available. I think this is not sufficient. For the most "exotic" assignments the authors should provide fully assigned spectra to support their conclusions. I would e.g. love to see the assigned MS/MS spectrum of S2H3N10F5 now indicated in Figure 2. Also for the small number of additional, de novo assigned glycopeptides, a display of the assigned spectra may help to support the claims.**

We have now provided the PRIDE database ID for each glycan composition in the respective tables to make it easier to access the mass spectrometric data. The spectra and fragments were also previously accessible in the database. For clarity and simplicity, we have removed the de novo compositions from this study.

**2. Another aspect of concern is the use of "Max fold change". I think one can safely agree that "Max fold change" is not a conventional way of assessing differences between groups, and is not the most meaningful parameter. Obviously, I am not interested in seeing the maximum difference between groups, but the differences of medians or means in these cross-sectional comparisons. Displaying such averages and ranges (e.g. interquartile ranges, or standard deviations) would make the paper biologically way more meaningful.**

We have now removed the maximum fold changes and replaced them with fold changes calculated using the difference of the mean abundance of each glycopeptide between the groups compared.

**3. As a reader I would need more relevant information in the supplemental tables: what is the score column? Were the p-values corrected? If so, in which manner, with which factor? Which significance threshold was applied?**

The glycopeptide scores in the supplemental tables are calculated as a negative logarithm of the probability that a random set of fragments would have as many or more shared peaks with the measured spectrum as the ranked glycopeptide. The probability that the random spectra have more or equal shared peaks than the glycopeptide spectrum is calculated using binomial distribution. The glycopeptide scores therefore provide an indication of the quality of the identification. We have now referred to the study explaining this in detail in the Material and methods section.

The p-values provided in the tables in the manuscript are uncorrected, with p-values of <0.05 being considered significant. We also used the Benjamini-Hochberg procedure to control the false-discovery rate and provide information regarding if the adjusted p-values were significant or not in the supplementary tables.

**4. For some glycopeptides the antennary fucoses were assigned to galactoses, for other to N-acetylglucosamine. I would love to see the data that support this assignment, in the form of some exemplary MS/MS spectra. I would be particularly interested to know whether the authors saw diagnostic B ions such as fucose-hexose, that would substantiate such a claim.**

The MS/MS spectra of all fucosylated glycopeptides were manually examined in order to see if, in the case of fucose residues assigned to the core, we could identify primarily and secondarily peptide-HexNAc-fucose ions so that monosaccharides instead of HexNAc residues could be assigned to the N-core, as only one fucose can be assigned to the N-core. In the case of fucose residues assigned to branches, we looked for fucose-hexose-HexNAc (FHN) ions.

In cases where fucose residues were only assigned to the core, no FHN ions were present in any of the spectra. In cases where fucose residues were assigned to branches, the software identified diagnostic FHN ions in 28 cases but missed 3 cases. In all cases, the final glycan composition used is the one with the best fit as calculated by fitting the total mass of the glycan composition, all the available intact peptide-glycan fragments, glycan fragments, and peptide-partial glycan fragments to the peptide whose sequence was assigned to that glycan. However, we would like to mention that wherever mentioned, we have only discussed proposed glycan structures.
